# Molecular targeted therapy for the treatment of gastric cancer

**DOI:** 10.1186/s13046-015-0276-9

**Published:** 2016-01-04

**Authors:** Wenting Xu, Zhen Yang, Nonghua Lu

**Affiliations:** Department of Gastroenterology, The First Affiliated Hospital of Nanchang University, Nanchang, Jiangxi 330006 China

**Keywords:** Molecular targeted therapy, Gastric cancer, Monoclonal antibody, Tyrosine kinase inhibitor

## Abstract

Despite the global decline in the incidence and mortality of gastric cancer, it remains one of the most common malignant tumors of the digestive system. Although surgical resection is the preferred treatment for gastric cancer, chemotherapy is the preferred treatment for recurrent and advanced gastric cancer patients who are not candidates for reoperation. The short overall survival and lack of a standard chemotherapy regimen make it important to identify novel treatment modalities for gastric cancer. Within the field of tumor biology, molecular targeted therapy has attracted substantial attention to improve the specificity of anti-cancer efficacy and significantly reduce non-selective resistance and toxicity. Multiple clinical studies have confirmed that molecular targeted therapy acts on various mechanisms of gastric cancer, such as the regulation of epidermal growth factor, angiogenesis, immuno-checkpoint blockade, the cell cycle, cell apoptosis, key enzymes, c-Met, mTOR signaling and insulin-like growth factor receptors, to exert a stronger anti-tumor effect. An in-depth understanding of the mechanisms that underlie molecular targeted therapies will provide new insights into gastric cancer treatment.

## Background

Gastric cancer is a common malignancy of the digestive system and is the second most common cause of cancer-related death [1]. Over 1,000,000 new cases occur each year, of which more than 70 % are diagnosed in developing countries, particularly in East Asia [2]. Although surgery is the primary method for gastric cancer treatment, the majority of patients exhibit advanced disease at the time of diagnosis, which limits the effectiveness of surgery. Chemotherapy is appropriate for these patients. However, the objective response rate is only 20–40 %, and the median overall survival (OS) time is only 6–11 months following chemotherapy [3]. Moreover, the serious side effects of chemotherapy cannot be ignored.

**Table 1:** Characteristic of molecular targeted agents for gastric cancer treatment

Agent	Type	Target(s)	Current prospects for gastric cancer therapy
Trastuzumab	Recombinant humanized mAb	HER-2	Approved by FDA
Ramucirumab	Humanized mAb	VEGFR	Approved by FDA
Sorafenib	Tyrosine kinase inhibitor	VEGF, PDGF	Phase II or III clinical trials are ongoing
Marimastat	Inhibitor	MMPs	Phase II or III clinical trials are ongoing
Erlotinib	Tyrosine kinase inhibitor	EGFR	Phase II clinical trials are ongoing
Foretinib	Inhibitor	c-Met, KDR VEGFR2	Clinical trials are ongoing
Bevacizumab	Humanized mAb	VEGF	Individualized treatment [47]
Pertuzumab	Recombinant humanized mAb	HER-2	A Phase III clinical trial is ongoing [23]
Sunitinib	Tyrosine kinase inhibitor	VEGF, PDGF, KIT, FLT-3, RET	Promising [50-52]
Bortezomib	Proteasome inhibitor	NF-κB	Promising [59]
Rilotumumab	Inhibitor	c-Met	Promising [69]
Everolimus	Inhibitor	mTOR	Not satisfactory [75]
Lapatinib	Tyrosine kinase inhibitor	EGFR, HER-2	Satisfactory for a specific population [30, 31]
Gefitinib	Tyrosine kinase inhibitor	EFGR	Limited efficacy [26]
Cetuximab	Humanized mAb	EFGR	Not satisfactory [17]
Panitumumab	Humanized mAb	EFGR	Not satisfactory
Flavopiridol	Semi-synthetic flavonoid inhibitor	CDK	Not satisfactory (Phase II clinical trial failed)
Figitumumab	Humanized mAb	IGFR-IR	Phase I clinical trials are ongoing

*Abbreviations*: *mAb* monoclonal antibody, *EGF* epidermal growth factor, *EGFR* epidermal growth factor receptor, *FDA* Food and Drug Administration, *MMP* matrix metalloproteinase, *VEGF* vascular endothelial growth factor, *PDGF* platelet-derived growth factor, *RET* rearranged during transfection, *FLT3* FMS-like tyrosine kinase-3 receptor, *CDK* cyclin-dependent kinase

**Table 2:**
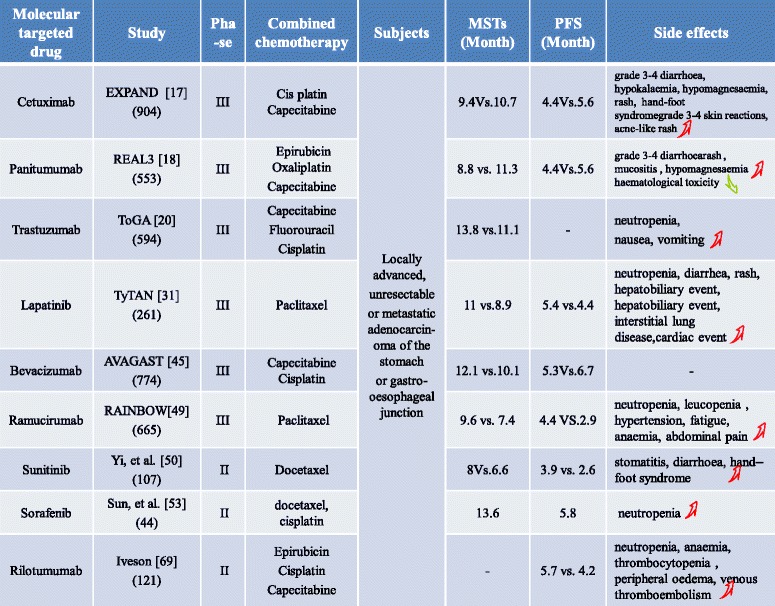
Evaluation of combination chemotherapy for gastric cancer treatment

**Fig. 1:**
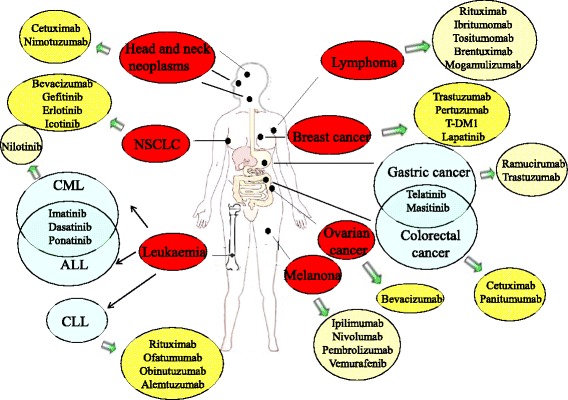
Molecular targeted agents approved by the FDA for different cancers. Abbreviations: CML, Ph + chronic myeloid leukemia; ALL, lymphoblastic leukemia; NSCLC, non-small cell lung cancer; CLL, chronic lymphocytic leukemia

As a result of the rapid advancements in the field of tumor biology, attention has been focused on the new modality of molecular targeted therapy for advanced cancer. Molecular targeted inhibitors effectively regulate overexpressed molecules in tumor cells and the signaling pathways that are closely associated with tumorigenesis, thereby modulating the biological behavior of tumor cells [4]. Molecular targeted therapy not only improves the specificity and selectivity of anti-cancer therapy but also avoids non-selective toxicity and resistance. A substantial number of molecular targeted drugs have been approved by the Food and Drug Administration (FDA) for clinical use (Fig. 1). A comprehensive understanding of the theoretical basis of molecular targeted therapy will facilitate breakthroughs in the clinical treatment of gastric cancer.

## Agents that target epidermal growth factor receptor (EGFR)

**Fig. 2:**
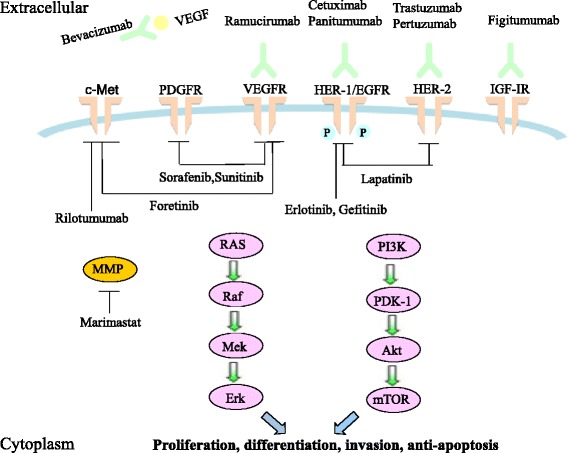
Mechanism of molecular targeted therapy in gastric cancer. Molecular targeted therapy acts on various mechanisms of gastric cancer, such as EGFR, angiogenesis, PDGF, IGF-1R, key enzymes or c-Met, and thus activates related signaling pathways to promote cell proliferation, differentiation, invasion and suppress apoptosis. Abbreviations: VEGFR, vascular endothelial growth factor receptor; PDGFR, platelet-derived growth factor receptor; IGF-1R, insulin-like growth factor 1 receptor; FGFR, fibroblast growth factor receptor

EGFR is a transmembrane glycoprotein that is composed of 1186 amino acids. The EGFR family includes four members: HER-l (EGFR), HER-2 (Neu), HER-3 and HER-4. HER-2 and HER-3 bind to other EGFR family members to form a heterodimer. For example, HER-2 binds EGFR, and the kinase activity of HER-2 subsequently phosphorylates the heterodimer, which leads to phosphatidylinositol 3-kinase (PI3K)/Akt and Ras/MEK signaling pathway activation [5]. These pathways promote cell proliferation, differentiation and invasion and suppress apoptosis (Fig. 2). An abnormally high expression of EGFR and HER-2 has been identified in gastric cancer cells, colorectal cancer cells and esophageal squamous cell carcinoma cells [6–8]. The expression levels of HER-1 and HER-2 are positively correlated with the depth of tumor invasion and negatively correlated with the degree of tumor differentiation and survival duration. Therefore, drugs that target EGFR and HER-2 are expected to improve the therapeutic efficacy of gastric cancer treatments.

### Anti-EGFR monoclonal antibodies

Cetuximab (Cetuximab, C225) is a humanized IgG1 monoclonal antibody that specifically binds to the extracellular domain of EGFR. This antibody competitively inhibits the binding of EGFR to its natural ligands and blocks the ligand-induced phosphorylation of the tyrosine kinase domain of EGFR. Cetuximab downregulates the expression of cell surface receptors and weakens receptor-related signaling. Cetuximab also kills tumor cells via antibody-dependent cellular cytotoxicity [9]. Since cetuximab was introduced to the market in 2004 [10], numerous phase II clinical studies have assessed the efficacy and safety of its combination with other chemotherapy regimens, including FOLFIRI, docetaxel/cisplatin, FOLFOX, and XELOX [11–15]. These clinical trials have identified a tumor response rate of 41.2–52.3 % and a median OS time of 5.4–16 months for patients with advanced gastric cancer. Based on data from multiple studies, there is no significant difference in the results between first-line treatment with a combination treatment of cetuximab and other chemotherapy regimens and the use of a single-agent chemotherapeutic regimen; however, the former treatment represents an alternative choice for first-line treatment because of its relatively lower toxicity. As a second-line treatment, combination regimens including cetuximab are clearly advantageous in terms of reduced toxicity. However, whether this regimen can be regarded as the standard regimen depends on economic factors. Many Phase II and III clinical trials to investigate the effects of combination regimens including cetuximab are ongoing [16]. Cetuximab in combination with capecitabine and cisplatin in advanced esophagogastric cancer (EXPAND), a phase III clinical trial, evaluated the efficacy of cetuximab in combination with capecitabine and cisplatin as a first-line treatment for advanced gastric cancer [17]. However, no significant difference in the median OS time or median progression-free survival (PFS) was identified between the experimental and control groups (4.4 vs 5.6 months, respectively, *p* = 0.32 and 9.4 vs 10.7 months, *p* = 0.95, respectively), although the incidence of adverse reactions was increased in the experimental group compared with the control group. Multivariate analysis indicated that mutations in the Kirsten rat sarcoma viral oncogene homolog (KRAS) and PIK3CA were poor prognostic factors. In colon cancer, the efficacy of cetuximab was increased in patients who carried wild-type KRAS; patients who carried the mutant KRAS were resistant to cetuximab treatment. However, the relationship between KRAS expression and treatment efficacy in gastric cancer patients has not been established.

In contrast to cetuximab, panitumumab is a completely humanized monoclonal antibody. Panitumumab is beneficial for colorectal cancer patients who have failed FOLFOX treatment. In REAL3, a phase II/III clinical trial, treatment with panitumumab combined with modified epirubicin, oxaliplatin, and capecitabine did not improve the condition of patients with esophageal, gastroesophageal junction or gastric cancer or with undifferentiated carcinoma [18]. The median OS time using this regimen was significantly shorter than the standard regimen of epirubicin, oxaliplatin, and platinum (median OS time of 11.3 months); furthermore, the median PFS was shorter in the experimental group compared with the control group (6.0 vs 7.4 months, respectively, *p* = 0.068). The tendency toward shorter survival may have been a result of inadequate chemotherapy drug doses, accelerated cancer progression after drug withdrawal or an inability to continue therapy because of the deterioration of the host’s condition. The failure of the EXPAND and REAL3 trials suggested that EGFR may not be the primary oncogenic driver in advanced gastric cancer. Therefore, the identification of predictive markers of anti-EGFR treatment outcome to determine the population that would most likely benefit from this therapy is crucial for therapeutic efficacy.

### Anti-HER-2 monoclonal antibodies

Trastuzumab is a recombinant, humanized IgG1 monoclonal antibody that binds to the HER-2 receptor to eliminate or reduce receptor activity, which thereby weakens subsequent signaling events that involve molecules such as protein kinase B (PKB) and signal transducer and activator of transcription 3 (STAT3). In addition, trastuzumab induces antibody-dependent cytotoxicity by increasing the expression of the cyclin-dependent kinase (CDK) inhibitor p27, which causes the downregulation of cell cycle proteins and cell cycle disorders [19]. The outcomes of the ToGA trial by Bang et al. [20] comprised the initial evidence to demonstrate that combination chemotherapy with trastuzumab improves the survival rate of HER-2-positive advanced gastric cancer patients. The study defines the standard for HER-2 positive as “+++” via immunohistochemistry (IHC) or “+” via fluorescence in situ hybridization (FISH). The high expression of HER-2 was IHC (+++) or IHC (++) and FISH (+), and the low expression of HER-2 was FISH (+) and IHC (− or +). Overall, 584 cases were included and randomly divided into two groups based on treatment with trastuzumab plus chemotherapy (294 cases) and chemotherapy (290 cases). The results demonstrated that the efficacy endpoint in the trastuzumab plus chemotherapy group significantly improved: 47 vs 35 %, respectively, for total efficiency (*p* = 0.0017); 13.8 vs 11.1 months, respectively, for the median OS time (*p* = 0.0046); and 6.7 vs 5.5 months, respectively, for PFS (*p* = 0.0002). Further analysis indicated that in the 446 cases with high expression of HER-2, the median OS time was significantly increased in the trastuzumab plus chemotherapy compared with the chemotherapy group (16.0 vs 11.8 months, respectively). This trial prolonged the OS of patients beyond one year, and the quality of life of the patients with advanced gastric cancer was greatly improved. These findings highlight the advantages of individualized treatment. Despite such impressive results, many noteworthy issues in clinical applications remain considering the results of the subgroup analysis: only patients with IHC (+++) or IHC (++)/FISH (+) benefitted from the combination therapy, while the cases of IHC (−) or IHC (+)/FISH (+) did not show benefits. Furthermore, the combination was only effective for intestinal gastric cancer. There was no significant difference in Asian patients between the two groups. Given the evidence regarding the first-line treatment, the European Medicines Agency (EMA) and the U.S. FDA approved trastuzumab combined with either capecitabine or 5-FU and DDP to treat metastatic gastric cancer or gastroesophageal junction cancer in 2010. Furthermore, the HELOISE trial is investigating the optimal dose of trastuzumab in these patients.

Pertuzumab is a recombinant humanized monoclonal antibody that binds to the extracellular domain of HER-2 and directly suppresses the dimerization of HER-2, which thereby inhibits downstream signaling pathways. The primary difference between pertuzumab and trastuzumab is that the trastuzumab-induced inhibition of ligand-induced dimerization is dependent on HER-2 expression levels, which suggests that a broader range of individuals will benefit from pertuzumab [21]. To date, there are no clinical reports regarding the use of pertuzumab in the treatment of gastric cancer. However, a preclinical study demonstrated that pertuzumab combined with trastuzumab enhanced the anticancer effect in an HER2-positive gastric cancer xenograft model [22]. A double-blind, placebo-controlled, randomized clinical study, JACOB, is ongoing to evaluate the efficacy and safety of pertuzumab combined with trastuzumab and cisplatin and 5-FU/capecitabine treatment in HER2-positive metastatic gastric cancer or gastroesophageal junction cancer [23]. The study is expected to include 780 patients from 35 countries.

### Tyrosine kinase inhibitors (TKIs) of EGFR/HER-2

TKIs competitively antagonize the binding of ATP to a TKD, which thus inhibits the autophosphorylation of receptor tyrosine kinases and blocks the activation of EGFR-mediated signaling pathways, ultimately leading to the inhibition of tumor cell proliferation. TKIs provide more apparent benefits to patients with mutant *EGFR* compared with patients with translocated wild-type *EGFR* [24]. Gefitinib was the first TKI used to treat cancer. Gefitinib exhibits biological activity in tumor cells and increases the sensitivity of these cells to radiation [25]. However, the efficacy of gefitinib in the treatment of gastroesophageal junction adenocarcinoma is not ideal [26]. This limited efficacy may result from rare *EGFR* mutations, particularly gefitinib-related mutations, such as delE746-A750 or L858R, in esophagogastric junction adenocarcinoma [27]. Therefore, gefitinib is not primarily recommended for gastric cancer treatment.

Erlotinib (Tarceva) is another small-molecule tyrosinase inhibitor. The Southwest Cancer Cooperative Group conducted a phase II clinical trial (SWOG 0127) and reported the effectiveness of erlotinib for the treatment of gastroesophageal junction adenocarcinoma [28].

In addition, several tyrosinase inhibitors, such as lapatinib, target both EGFR and HER-2. These inhibitors not only prevent the autophosphorylation and activation of these receptors in tumor cells but also bind to EGFR or HER-2 dimers to inhibit downstream signaling pathways [29]. TRIO-013/(LOGiC), a phase III clinical trial, compared the efficacy of capecitabine and oxaliplatin with and without lapatinib to treat HER-2-positive advanced gastric, esophageal junction and gastroesophageal cancers [30]. Lapatinib did not significantly improve the median OS time compared with chemotherapy alone. Despite the increase in the median OS time and the objective response rate in the experimental group compared with the control group, the incidence of diarrhea and skin toxicity was substantially higher in the former compared with the latter group. However, a subgroup analysis indicated that patients <60 years of age and Asian patients greatly benefited from the addition of lapatinib. Another phase III clinical trial, TyTAN, determined that lapatinib combined with paclitaxel as a second-line regimen for advanced gastric cancer in patients who exhibited amplification of HER-2 (FISH-positive) did not significantly alter the median OS time (11 vs 8.9 months, respectively) or the mean PFS compared with paclitaxel alone (5.4 vs 4.4 months, respectively) [31]. TyTAN demonstrated that lapatinib prolonged the survival of patients who received this second-line treatment for advanced gastric cancer; however, this conclusion was specific to HER-2-positive patients. The efficacy of lapatinib for gastric cancer may not be as beneficial as trastuzumab. This discrepancy may be attributed to individual differences in drug metabolism and bioavailability, as well as lapatinib-related treatment resistance. Studies have demonstrated that lapatinib resistance may be associated with secondary HER-2 mutations, MET overexpression, and PTEN deletion [32–34]. However, in mainland China, patients exhibited an increased median OS time and median PFS when administered lapatinib and paclitaxel compared with paclitaxel alone [31]. The subgroup analysis demonstrated that lapatinib may provide a survival benefit to Chinese patients. Thus, additional prospective studies of Asian patients with HER-2-positive advanced gastric cancer are warranted.

## Agents that target vascular endothelial growth factor (VEGF)

Cancer is a vascular-dependent disease. When the tumor volume reaches 2 mm^3^, the tumor cells become hypoxic and secrete a broad range of factors to promote tumor angiogenesis, growth and invasion. Therefore, interventions that target tumor angiogenesis have become a primary strategy for cancer therapy.

VEGF is one of the most important cytokines in the induction of tumor angiogenesis. VEGF induces tumor angiogenesis by promoting endothelial cell proliferation and increasing vascular permeability. VEGF expression is commonly high in gastric cancer tissues and is related to the invasiveness, clinical stage and prognosis of gastric cancer [35]. Anti-VEGF antibodies and VEGF inhibitors are expected to block angiogenesis and downstream signaling, which thereby decrease tumor blood flow and nutrient supply and increase vascular permeability to promote drug penetration into the tumor.

### Anti-VEGF monoclonal antibodies

Bevacizumab is a humanized anti-VEGF monoclonal antibody that specifically binds VEGF, which inhibits the binding of VEGF to the VEGF receptor (VEGFR) and blocks the activation of tyrosine kinase signaling pathways. These effects suppress the proliferation of endothelial cells and inhibit angiogenesis. Humanization is beneficial for extending the half-life and reducing the immunogenicity of a therapeutic antibody. Bevacizumab, which highly specifically recognizes and binds to VEGF, was the first FDA-approved anti-VEGF monoclonal antibody for cancer treatment. It has been acknowledged for clinical use to treat colorectal cancer, non-small cell lung cancer (NSCLC), breast cancer, renal cell carcinoma, ovarian cancer and glioblastoma [36–44]. To assess its value as a first-line treatment for late-stage gastric cancer, a randomized, double-blind phase III clinical trial, referred to as the AVAGAST Study, was conducted. In this study, the OS increased from 10.1–12.1 months in the control group, the median PFS increased from 5.3 –6.7 months, and the overall response rate improved (37 vs 46 %, respectively); however, these differences were not significant [45]. Adverse reactions to bevacizumab, such as embolic disease and gastric perforation, should not be overlooked [46]. Van Cutsem et al. [47] evaluated whether angiogenesis markers predicted the effects of bevacizumab and determined that the efficacy of bevacizumab was related to the baseline expression of VEGF-A and Neuropilin-1. In a non-Asian population, the median OS time was longer in patients with a high level of VEGF-A expression and a low level of Neuropilin-1 expression. These findings suggest that the future development of gastrointestinal cancer treatments must include the identification of appropriate biomarkers for individualized treatment.

### Anti-VEGFR monoclonal antibodies

Ramucirumab is a completely humanized monoclonal antibody against VEGFR. A randomized phase III study of advanced gastric cancer patients who had failed first-line treatment demonstrated that best supportive care plus ramucirumab significantly improved the OS, PFS, and disease control rate compared with the control group [48]. Another phase III clinical study demonstrated that the combination of ramucirumab and chemotherapy markedly increased the OS, PFS and disease control rate for advanced gastric cancer [49]. Therefore, based on the results of these two important phase III clinical trials, the EMA and the U.S. FDA approved ramucirumab as the second-line therapy for gastroesophageal junction and chemotherapy-failed gastric adenocarcinomas, either alone or in combination with paclitaxel, in 2014. However, its primary adverse reactions, such as neutropenia, leukopenia, hypertension and decreased strength, cannot be ignored.

### TKIs

Sunitinib is an oral multi-targeted TKI. Its mechanism of action involves the inhibition of the tyrosine kinase activity of VEGF, platelet-derived growth factor receptor-β, KIT (stem cell factor receptor), FMS-like tyrosine kinase-3 receptor (FLT-3) and rearranged during transfection (RET); it consequently specifically blocks signal transduction associated with these kinases to exert anti-tumor effects. In a phase II clinical study, the combination of sunitinib and docetaxel improved the objective remission rate (41.1 vs 14.3 %, respectively) in patients with metastatic gastric cancer compared with docetaxel alone [50]. Various phase І and II clinical trials have confirmed the safety and tolerability of sunitinib combined with traditional chemotherapies; however, it clinical significance requires further investigation in randomized controlled trials [50–52]. Sorafenib is another multi-targeted TKI. Sorafenib directly suppresses the Raf-MEK-ERK signaling pathway and indirectly reduces VEGFR activity, which thus inhibits tumor growth. Sorafenib is beneficial for more than 20 types of malignant cancer and is associated with few side effects. A phase II clinical trial that included 44 patients with advanced gastric cancer indicated that sorafenib combined with docetaxel and cisplatin was associated with a 5.8 month median PFS and a 13.6 month median OS time [53]. Phase П clinical trials regarding the efficacy of orafenib combined with oxaliplatin and capecitabine are ongoing. Additional clinical trials are necessary to determine the efficacy of these anti-VEGF TKIs in the treatment of advanced gastric cancer.

## Immuno-checkpoint blockade

The development of a monoclonal antibody targeting immuno-checkpoints has attracted substantial attention in recent years. It can induce sustained tumor remission and is effective in treating various tumors. Immuno-checkpoints are inhibitory signaling pathways in the immune system that not only regulate the sustainability and strength of the immune response in peripheral tissue to avoid tissue damage but are also involved in maintaining tolerance to self-antigens. Targeting these inhibitory signaling pathways to inhibit T cell activity is a key mechanism to avoid tumor escape from immunologic cytotoxicity. Monoclonal antibodies targeted at PD-1, PD-L1 and CTLA-4 have been gradually implemented in clinical research. Moreover, the synergistic effects of various immunosuppressors are expected to represent an effective treatment mode. Duraiswamy et al. used PD-1 and CTLA-4 antibodies to block multiple immuno-checkpoints, which led to the enhancement of T cell reactivity. In the near future, a combined antibody targeted at PD-1, PD-L1 and CTLA-4 will be developed for the treatment of gastric cancer [54].

## Agents that act on the tumor cell cycle

Cancer is a disorder of cell cycle regulatory mechanisms. There are three main types of molecules involved in cell cycle regulation: cyclins, CDKs and CDK inhibitors (CKIs). CDKs bind to cyclins, which facilitates the crossing of restriction points by cells during cell cycle progression. CDKs also combine with CKIs to inhibit cell cycle progression or induce apoptosis [55]. Therefore, CKIs likely induce cell cycle arrest at certain phases.

Flavopiridol is a semi-synthetic flavonoid CKI and was the first cell cycle inhibitor evaluated in a clinical trial. Flavopiridol extensively suppresses messenger RNA translation by blocking the transport of transcripts to ribosomes, which leads to the cessation of cell proliferation-related protein expression [56]. However, flavopiridol failed to exert the desired effect on gastric cancer as a result of low efficacy and serious adverse reactions [57]. Considering its poor anti-tumor activity as a single agent, further investigation of its use combined with other chemotherapeutic agents is necessary.

## Agents that act on tumor cell apoptosis

Tumor cells are typically characterized by enhanced proliferation, impaired differentiation and inhibited apoptosis. Consequently, the promotion of apoptosis is an important topic in cancer therapy. Tumor necrosis factor-related apoptosis-inducing ligand (TRAIL) selectively induces the apoptosis of multiple types of tumor cells. Gastric cancer cells often exhibit resistance to TRAIL-induced apoptosis; however, various chemotherapeutic agents enhance the sensitivity of gastric cancer cells to TRAIL [58]. Therefore, there is a potentially substantial benefit to combining TRAIL with chemotherapies for the treatment of gastric cancer.

NF-κB belongs to the NF-κB/Rel protein family. NF-κB expression positively correlates with the degree of malignancy and negatively correlates with cancer prognosis. Bortezomib, a proteasome inhibitor, specifically inhibits the chymotrypsin activity of the 26S proteasome, which thereby inhibits the activation of the NF-κB signaling pathway. A phase II study demonstrated that the bortezomib treatment efficacy was 66 %, which suggests that bortezomib is a feasible option for relapsed/refractory cancer [59].

## Progress in other related fields

### Matrix metalloproteinase (MMP) inhibitors

MMPs include a series of proteolytic enzymes that participate in the degradation and destruction of the extracellular matrix and the basement membrane. The abnormal expression of MMPs promotes local tumor invasion and spread. High expression of MMP-2, MMP-9, MMP-14 and MMP-21 is associated with the progression and poor prognosis of gastric cancer [60–63]. The MMP inhibitor marimastat (BB-2516, TA-2516) exhibits anti-tumor activity in gastric cancer. Advanced gastric cancer patients also benefit from marimastat because of its low hematological toxicity, which confirms the clinical value of marimastat for gastric cancer patients [64].

### c-Met signaling pathway inhibitors

c-Met is a membrane receptor tyrosine kinase capable of binding to HGF and activating the HGF/c-Met signaling pathway, which thereby regulates the proliferation and migration of tumor cells [65]. Furthermore, HGF/c-Met signaling may block the activation of β4-integrin, CD44 and non-kinase molecules, which are closely related to an enhanced capacity for invasion and angiogenesis [66]. Because of its position at the intersection of multiple signaling pathways that are closely related to tumor formation and metastasis, c-Met has become a promising new target as modifying its activity may simultaneously interfere with all relevant pathways [67]. c-Met overexpression has been observed in gastric cancer patients [68]. A phase II clinical trial demonstrated that a combination regimen that contained rilotumumab, a c-Met inhibitor, provided a survival benefit [69]. Currently, the efficacy of rilotumumab has been demonstrated for c-Met-positive gastric cancer or esophagogastric junction cancer in RILOMET-1, a phase III clinical study [70].

Foretinib is a new type of inhibitor of c-Met and VEGFR2/KDR. In 2009, Kwak et al. reported good efficacy and safety of foretinib in a phase I clinical study at an ASCO meeting [71]. Based on reliable evidence, the FDA approved the drug to directly enter phase III trials for the treatment of NSCLC without a phase II trial. However, few studies have investigated the use of foretinib in gastric cancer; thus, further research is necessary.

### Therapies targeting mTOR

mTOR belongs to the PI3K-related kinase family, which primarily regulates cell growth, cell proliferation, the cell cycle, and other physiological functions via the PI3K/Akt/mTOR signaling pathway [72]. The expression of phosphorylated mTOR is a prognostic factor for gastric cancer that negatively correlates with cancer prognosis [73]. Everolimus prevents the phosphorylation of p70S6K and 4E-BP1 mediated by mTOR, which results in G0/G1 arrest [74]. However, a phase III clinical study of everolimus in advanced gastric cancer patients who failed previous chemotherapy treatment demonstrated that there was no significant efficacy of everolimus in gastric cancer [75].

### Cyclooxygenase-2 (COX-2) inhibitors

COX is an important rate-limiting enzyme in the conversion of arachidonic acid to prostaglandins. COX-1 and COX-2 are two COX isoenzymes. COX-2 is barely expressed under physiological conditions; however, it is highly expressed in gastric cancer. COX-2 is involved in tumorigenesis and cancer development via the promotion of cell proliferation, suppression of apoptosis, and induction of tumor angiogenesis. COX-2 inhibitors have been demonstrated to exert anti-cancer effects on gastric cancer cells; however, precise clinical trial data are lacking [76]. Importantly, the gastrointestinal adverse effects of COX inhibitors limit their widespread clinical application.

### Insulin-like growth factor receptor (IGF-IR) inhibitors

IGF-IR is a transmembrane tyrosine kinase receptor, which is activated after binding to IGF-1 and IGF-2. IGF-IR plays a key role in malignant transformation, angiogenesis, metastasis, and anti-apoptosis. Figitumumab (CP-751871) is a completely humanized IgG2 monoclonal antibody against IGF-IR. Phase І and II clinical studies have confirmed the efficacy of figitumumab on Ewing’s sarcoma and NSCLC; however, a phase Ш clinical study of NSCLC was terminated halfway due to no reaching the endpoint [77–79]. Studies of this agent in gastric cancer treatment are in the clinical research stage.

## Conclusions and challenges

**Fig. 3:**
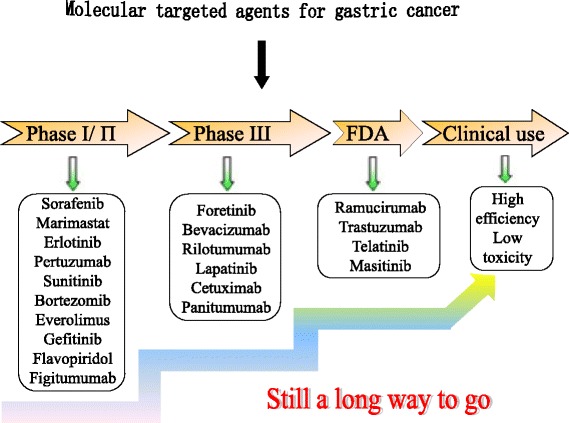
Evolution of molecular targeted agents for the treatment of gastric cancer

Although an increasing number of clinical studies has explored effect of targeted therapy alone or in combination with chemotherapy in the field of gastric cancer (Tables 1 and 2), its application in gastric cancer remains in its infancy compared with its successful use in colon, lung, and breast cancers. Targeted therapy for gastric cancer continues to face enormous challenges (Fig. 3) (1). Numerous phase II clinical trials have been performed; however, precise Phase III clinical trials are lacking. Additional in-depth Phase III clinical studies must be pursued to obtain sufficient evidence to support the use of targeted therapy for gastric cancer. (2) Targeting a single molecule has limited use for the treatment of gastric cancer because of the complex pathogenesis of the disease. Consequently, drugs that target a single molecule will be susceptible to a loss of efficacy soon after compensatory mechanism activation. Furthermore, it is difficult to target the entire tumor because subclones of gastric cancer cells exhibit different biological behaviors. This is only one of the primary reasons for the failure of single agents as a broad treatment for gastric cancer. Therefore, the development of multi-target drugs or the combination of targeted drugs with surgery, radiotherapy and chemotherapy may result in new opportunities for cancer treatment. (3) Individual differences create challenges; not all patients benefit from a new treatment modality. The detection of specific biomarkers or related genes will be required to develop individualized treatment. (4) The cost of targeted drugs remains one of the greatest obstacles to their widespread use in clinical practice and must decrease. Despite these challenges, we believe that an in-depth understanding of the molecular mechanisms that underlie tumor development will lead to breakthroughs in the targeted treatment of gastric cancer, thereby opening a new chapter for advanced gastric cancer.

## Abbreviations

ALL: lymphoblastic leukemia
CDK: cyclin-dependent kinase
CLL: chronic lymphocytic leukemia
CML: Ph + chronic myeloid leukemia
EGF: epidermal growth factor
EGFR: epidermal growth factor receptor
EMA: European Medicines Agency
FDA: Food and Drug Administration
FGFR: fibroblast growth factor receptor
FISH: fluorescence in situ hybridization
FLT3: FMS-like tyrosine kinase-3 receptor
IGF-1R: insulin-like growth factor 1 receptor
IHC: immunohistochemistry
KRAS: Kirsten rat sarcoma viral oncogene homolog
mAb: monoclonal antibody
MMP: matrix metalloproteinase
NSCLC: non-small cell lung cancer
OS: overall survival
PDGFR: platelet-derived growth factor receptor
PFS: progression-free survival
PKB: protein kinase B
RET: rearranged during transfection
STAT3: signal transducer and activator of transcription 3
TKI: tyrosine kinase inhibitors
TRAIL: tumor necrosis factor-related apoptosis-inducing ligand
VEGFR: vascular endothelial growth factor receptor
